# Bioactive Terpenes and Their Derivatives as Potential SARS-CoV-2 Proteases Inhibitors from Molecular Modeling Studies

**DOI:** 10.3390/biom11010074

**Published:** 2021-01-07

**Authors:** Lúcio Ricardo Leite Diniz, Yunierkis Perez-Castillo, Hatem A. Elshabrawy, Carlos da Silva Maia Bezerra Filho, Damião Pergentino de Sousa

**Affiliations:** 1 College of Nordeste da Bahia, Coronel João Sá 48590-000, Brazil; luciodiniz@yahoo.com.br; 2 Bio-Cheminformatics Research Group and Escuela de Ciencias Físicas y Matemáticas, Universidad de Las Américas, Quito 170125, Ecuador; yunierkis@gmail.com; 3 Department of Molecular and Cellular Biology, College of Osteopathic Medicine, Sam Houston State University, Conroe, TX 77304, USA; hxe007@SHSU.EDU; 4 Department of Pharmaceutical Sciences, Federal University of Paraíba, João Pessoa 58051-900, Brazil; carlosmaia1996@gmail.com

**Keywords:** natural products, terpenoid, plants, saponins, Middle East Respiratory Syndrome Virus, SARS-CoV, MERS-CoV, SARS-CoV-2, COVID-19, viruses

## Abstract

The coronavirus disease 2019 (COVID-19) pandemic is caused by a novel coronavirus; the Severe Acute Respiratory Syndrome Coronavirus-2 (SARS-CoV-2). Millions of cases and deaths to date have resulted in a global challenge for healthcare systems. COVID-19 has a high mortality rate, especially in elderly individuals with pre-existing chronic comorbidities. There are currently no effective therapeutic approaches for the prevention and treatment of COVID-19. Therefore, the identification of effective therapeutics is a necessity. Terpenes are the largest class of natural products that could serve as a source of new drugs or as prototypes for the development of effective pharmacotherapeutic agents. In the present study, we discuss the antiviral activity of these natural products and we perform simulations against the M^pro^ and PL^pro^ enzymes of SARS-CoV-2. Our results strongly suggest the potential of these compounds against human coronaviruses, including SARS-CoV-2.

## 1. Introduction

Coronaviruses (CoVs) are positive single-stranded (_+_ss) RNA viruses that are classified within the family Coronaviridae, order Nidovirales [1]. The infectious bronchitis virus (IBV) was the first-discovered CoV that caused an outbreak of respiratory illness in chickens in the 1930s [2]. Mouse hepatitis virus (MHV) and transmissible gastroenteritis virus (TGEV) were later discovered, in the 1940s, as animal CoVs that infect mice and pigs, respectively [3]. Human CoVs, HCoV-229E and HCoV-OC43, were identified in the 1960s as the causative agents of mild respiratory diseases that present as common cold [4]. Since then, five other HCoVs were identified at different times, including Severe Acute Respiratory Syndrome-CoV (SARS-CoV) in 2003, HCoV-NL63 in 2004, HCoV-HKU1 in 2005, Middle East Respiratory Syndrome-CoV (MERS-CoV) in 2012, and the most recently discovered Severe Acute Respiratory Syndrome-CoV-2 (SARS-CoV-2) in December 2019 [5,6]. Similar to HCoV-229E and HCoV-OC43, HCoV-NL63 and HCoV-HKU1 cause the common cold; however, SARS-CoV, MERS-CoV, and SARS-CoV-2 are responsible for severe respiratory illnesses that progress to pneumonia [5,6]. In the 2002–2003 outbreak, SARS-CoV infected 8098 people and resulted in 774 deaths (10% fatality rate) [7], whereas MERS-CoV infected 2506 and killed 862 individuals (35% fatality rate) [8]. Since December 2019 and as of today, SARS-CoV-2, the causative agent of the COVID-19 disease, has infected millions of people around the world and resulted in thousands of deaths [9]. The disease severity and high mortality associated with SARS-CoV, MERS-CoV, and SARS-CoV-2 necessitate the rapid discovery of effective antivirals.

CoV genomes consist of multiple genes that code for structural and nonstructural proteins [10]. The 5′ two-thirds of the genome consists of two open reading frames (ORF1a and ORF1b) that are translated into two polyproteins (pp1a and pp1ab), using a ribosomal frameshift sequence, once the viral RNA genome is in the host cell cytoplasm [10]. The polyproteins are internally processed by the viral proteases, 3-Chymotrypsin-like protease (3CL^pro^ or M^pro^) and papain-like protease 2 (PL^pro^) that are part of the two polyproteins [10]. The processing of polyproteins releases 16 viral nonstrucutral proteins that are important for viral replication, including the RNA-dependent RNA polymerase (RdRp), helicase, and 3′-5′ exoribonuclease [10].

The remaining one-third of the genome codes for structural proteins, which include spike (S), membrane (M), Envelope (E), and nucleocapsid (N) proteins, as well as nonstructural proteins that serve important roles in viral replication and pathogenesis [10]. The S protein forms the spikes on the viral particle that bind to cognate receptors (angiotensin-converting enzyme 2 (ACE2) for SARS-CoV and SARS-CoV-2, and dipeptidyl-peptidase 4 (DPP4) for MERS-CoV)) on host cells and mediate viral entry [11,12,13]. Following viral replication, M, E, and N proteins are believed to mediate the assembly and release of viral particles from host cells [10]. 

Several research groups have developed neutralizing monoclonal antibodies that target SARS-CoV, SARS-CoV-2, and MERS-CoV S proteins and block viral entry [14]. In addition, several antiviral small molecules that target viral proteins and different steps in the viral life cycle have been described as potential inhibitors for SARS-CoV, SARS-CoV-2, MERS-CoV [14]. However, there are no commercially available antiviral drugs specifically developed for the treatment of CoV infections as of today [15]. 

Terpenes are isoprene-based natural compounds that are characterized by high chemical diversity and a wide range of therapeutic effects [16]. This class of natural products has been a valuable source for the identification of novel therapeutic agents. Classical drugs with different therapeutic applications are terpenoids or terpenoid derivatives that are extracted from medicinal plants [17]. For example, artemisinin, a sesquiterpene lactone extracted from *Artemisia annua* L., is an important antimalarial drug that is widely used in the treatment of malaria [18]. The noted antineoplastic agent paclitaxel, a terpene isolated from the bark of the *Taxus brevifolia* Nutt., is one of the most commercially successful anticancer agents used in the treatment of different kinds of cancer [19]. Terpenophenolic compounds, cannflavin A and B extracted from *Cannabis* spp., have been tested for the treatment of multiple diseases, including cancer and neurological disorders [20]. Despite the wide range of pharmacologic activities of terpenes, more than 80,000 natural terpenes might be potentially screened for therapeutic applications. Therefore, the present study aimed to discuss the anti-SARS-CoV-2 potential of this chemical class via analysis of the tests performed against several human coronaviruses and molecular docking in possible therapeutic targets related to this virus.

## 2. Methodology

The present study was carried out based on the literature review of terpenes and human coronavirus. The search, performed in the PubMed database, concerning studies published until March 2020, used the following keywords: coronavirus, terpenes, Middle East Respiratory Syndrome Virus, 229E, NL63, OC43, HKU1, SARS-CoV, MERS-CoV. or SARS-CoV-2 (2019-nCoV or COVID-19). The scientific publications on terpenes and derivatives against human coronaviruses were selected from studies published in English and discussed in this manuscript.

### 2.1. Molecular Docking

The crystal structures of the SARS-CoV-2 Main protease (M^pro^) and Papain-like protease (PL^pro^) were obtained from the Protein Data Bank database [21]. The structures of M^pro^ in complex with an α-ketoamide inhibitor (PDB code 6Y2G) [22] and that of PL^pro^ in complex with a peptide inhibitor (PDB code 6WX4) [23] were selected for modeling studies. One three-dimensional conformer was generated for each ligand, and am1-bcc partial atomic charges were added to them using OpenEye’s Omega [24] and Molcharge [25], respectively.

Molecular docking was performed with the Gold software [26] following the protocol described in our previous research [27,28]. The inhibitors cocrystallized with the enzymes were used as a reference for defining the binding pockets. Primary docking of each compound was performed with the CHEMPLP scoring function to generate 30 docking solutions. Each of these ligand poses were then rescored with the GoldScore, ChemScore, and ASP scoring functions. The most probable binding modes of each compound to the investigated receptors were selected according to the consensus scoring protocol previously described [27,28]. Any ligand conformation with a consensus score greater than 1 was selected for further analyses

### 2.2. Molecular Dynamics and Estimation of the Free Energies of Binding

MD simulations and the estimation of the free energies of binding were carried out with Amber 18 [29]. For MD simulations for M^pro^ we set up with one ligand present on each of the two active sites present in the dimer. These calculations proceed as previously described [30,31]. In summary, all the ligand–receptor complexes selected after the molecular docking calculations underwent the same modeling process. This protocol included systems preparation, energy minimization, equilibration, and production runs. All MD simulations took place in explicit solvent.

The equilibrated systems were used to seed five short (2 ns) production runs, each of which were initialized with different random initial atomic velocities. The free energies of binding of the ligands to M^pro^ and PL^pro^ were estimated with the MM-PBSA method as implemented in Amber 18. For this, 100 MD snapshots (one every 100 ps) were evenly extracted from the 10 ns of production MD simulations. For M^pro^, the ligand with the lowest ΔG of binding among those bound to the two monomers was analyzed.

## 3. Results and Discussion

The antiviral activity of plant terpenes has been evaluated on different CoVs. The studies show the anticoronavirus potential of several subtypes of terpenes isolated from different species, genera, and botanical families. For example, in 2012, Chang et al. [32] documented the significant inhibitory activity of friedelane-type triterpenoids, present in the ethanol extract from fresh leaves of *Euphorbia neriifolia* L., on HCoV-229E. Among the isolated friedelane derivatives, four terpenoid compounds exhibited higher anti-HCoV-229E activity, as indicated by the percentage of infected cell viability of 132.4, 80.9, 109.0, and 111.0%, shown by 3-β-friedelanol (**1**), 3-β-acetoxy friedelane (**2**), friedelin (**3**), and epitaraxerol (**4**), respectively. Of note, the antiviral activities of the tested compounds have been highly dependent on structural differences, as evidenced by significant variation of antiviral activity between 3-β-friedelanol and 3-α-friedelanol, which is the epimer with the inverted configuration on carbon-3. Also, the presence of acetyl group negatively affected the antiviral activity of 3-β-acetoxy friedelane, when compared to 3-β-friedelanol. Furthermore, 3-β-friedelanol showed antiviral activity against hepatitis B virus (HBV) through selective inhibition of HBeAg secretion [33].

Using the 2,3-bis[2 -methoxy-4-nitro-5-sulfophenyl]-5-[(phenylamino) carbonyl-2H-tetrazolium hydroxide] (XTT) assay, Cheng et al. [34] investigated the anti-HCoV-229E activity of oleanane-type triterpenoids derivatives named saikosaponin A (**5**), saikosaponin B_2_ (**6**), saikosaponin C (**7**), and saikosaponin D (**8**), which were isolated from *Bupleurum* spp., *Heteromorpha* spp., and *Scrophularia scorodonia*. All the tested saikosaponins demonstrated antiviral activity at concentrations of 0.25–25 µmol/L, with saikosaponin B_2_ showing the strongest activity (IC_50_ = 1.7 ± 0.1 µmol/L). The selectivity index (SI) values (CC_50_/IC_50_) of saikosaponin A (CC_50_ = 228.1 ± 3.8 µmol/L; SI = 26.6) and saikosaponin B_2_ (CC_50_ = 383.3 ± 0.2 µmol/L; SI = 221.9) indicated the lack of cytotoxic effects on target cells at concentrations that showed antiviral activity. In another study, saikosaponin B_2_ significantly inhibited HCoV-229E infection at 6 µmol/L following addition at different time points (pre-infection (-4 to -1 h), coinfection (0 h) and postinfection (1–4 h)). In addition, saikosaponin A inhibited the replication of three strains of influenza virus A, including H5N1 [35]. Saikosaponin B_2_ inhibited hepatitis C virus (HCV) entry into human hepatocytes [36], whereas saikosaponin C inhibited the viral replication of HBV in HepG2.2.15 cells [37]. Furthermore, saikosaponin D showed antiviral activity against herpes simplex virus and measles virus [38].

Park et al. [39] investigated the inhibition selectivity of seven isolated tanshinones from *Salvia miltiorrhiza* on SARS-CoV 3CL^pro^ and PL^pro^ that are synthesized and expressed in *Escherichia coli* as well as on the open reading frame (ORF) containing only the catalytic domains. In general, all of the isolated tanshinones exhibited noncompetitive dose-dependent inhibition of both cysteine proteases and acted as time-dependent inhibitors of PL^pro^. The results of the inhibition of SARS-CoV 3CL^pro^ and PL^pro^, respectively, as expressed by IC_50_ values, were tanshinone IIA (**9**) (IC_50_ = 89.1 ± 5.2 and 1.6 ± 0.5 µM), tanshinone IIB (**10**) (IC_50_ = 24.8 ± 0.8 and 10.7 ± 1.7 µM), methyl tanshinonate (**11**) (IC_50_ = 21.1 ± 0.8 and 9.2 ± 2.8 µM), cryptotanshinone (**12**) (IC_50_ = 226.7 ± 6.2 and 0.8 ± 0.2 µM), tanshinone I (**13**) (IC_50_ = 38.7 ± 8.2 and 8.8 ± 0.4 µM), dihydrotanshinone I (**14**) (IC_50_ = 14.4 ± 0.7 and 4.9 ± 1.2 µM), and rosmaraquinone (**15**) (IC_50_ = 21.1 ± 0.8 and 30.0 ± 5.5 µM). In fact, these terpenes exhibited a significant inhibitory effect on SARS-CoV cysteine proteases (3CL^pro^ and PL^pro^) and provide good scaffolds for further optimization and drug development against CoV infection. Tanshinone IIA also inhibited Tat-induced Human immunodeficiency virus-1 [40] and attenuated the viral myocarditis caused by coxsackievirus B3 [41], whereas cryptotanshinone showed anti-influenza A virus activity [42].

Ryu et al. [43] compared the potent SARS-CoV 3CL^pro^ competitive inhibitory enzymatic activities of celastrol (**16**) (IC_50_ = 10.3 µM), pristimerin (**17**) (IC_50_ = 5.5 µM), tingenone (**18**) (IC_50_ = 9.9 µM), and iguesterin (**19**) (IC_50_ = 2.6 µM), and four quinone-methide triterpenes isolated from *Tripterygium regelli*. According to the authors, the quinone-methide moiety in the A ring, together with the hydrophobic E ring, contribute to the potentiation of biological activity, which, in turn, seems to be a mode of specific inhibition of action by kinetic analysis. Other studies showed that celastrol inhibited dengue virus replication [44], hepatitis C virus [45], and human immunodeficiency virus [46]. In addition, pristimerin inhibited viral replication of the human cytomegalovirus in the human embryonic fibroblast cell line, MRC-5, without affecting cell growth [47].

In recent years, the anti-CoV activity of multiple compounds has been mainly attributed to the inhibition of the 3CL^pro^ and PL^pro^ [48,49]. Wen et al. [50] reported the inhibitory effects of fourteen terpenes (ferruginol (**20)**, dehydroabieta-7-one (**21**), sugiol (**22**), cryptojaponol (**23**), 8-β-hydroxyabieta-9(11),13-dien-12-one (**24**), 7-β-hydroxydeoxycryptojaponol (**25**), 6,7-dehydroroyleanone (**26**), 3-β,12-diacetoxyabieta-6,8,11,13-tetraene (**27**), pinusolidic acid (**28**), forskolin (**29**), cedrane-3-β,12 diol (**30**), α-cadinol (**31**), betulinic acid (**32**), betulonic acid (**33**)) isolated from *Chamaecyparis obtusa*, *Juniperus formosana*, and *Cryptomeria japonica*, at concentrations ranging from 3.3 to 10 µM, on SARS-CoV activity, using a Vero E6 cell-based cytopathogenic effect (CPE) assay. As indicated SI values of the terpenes tested, the most potent and safe inhibitors of SARS-CoV were ferruginol (SI = 58), 8-β-hydroxyabieta-9(11),13-dien-12-one (SI>510), 3-β,12-diacetoxyabieta-6,8,11,13-tetraene (SI = 193), betulonic acid (SI = 18), and 7-β-hydroxydeoxycryptojaponol (SI = 111). In addition, betulinic acid had antiviral effects against hepatitis C virus [51], hepatitis B [52], influenza A virus [53], and herpes simplex virus type-2 [54].

For a long time, the antiviral activities of glycyrrhizin (GL) (**34**), a triterpene saponin glycoside isolated from *Glycyrrhiza* spp., have been reported against different viruses including herpes, influenza A, human immunodeficiency virus-1, hepatitis B, and vesicular stomatitis virus [17,55,56]. Diverse mechanisms of action have been attributed to the antiviral activities of glycyrrhizin, such as reduction of the transport to the membrane, inhibition of fusion of the viral membrane, induction of interferon-gamma in T-cells, and reduction of viral latency [57]. The effect of glycyrrhizin in inhibiting the replication of SARS-associated coronavirus has also been recently investigated. In 2003, Cinatl et al. [58] assessed the antiviral activity of glycyrrhizin in Vero cells infected with two clinical isolates of CoV from patients with SARS-CoV admitted to the clinical center of Frankfurt University, Germany. Glycyrrhizin showed a significantly potent inhibition of SARS-CoV adsorption (EC_50_ = 600 mg/L; CC_50_ > 20,000 mg/L; SI = 33), penetration (EC_50_ = 300 mg/L; CC_50_ > 20,000 mg/L; SI = 33), and replication (EC_50_ = 2400 mg/L; CC_50_ >20,000 mg/L; SI = 8.3). According to the authors, the anti-CoV activity of glycyrrhizin is associated with the induction of nitrous oxide synthase, leading to an increase of the intracellular levels of nitric oxide and inhibition of the SARS-CoV replication in Vero cells. In accordance, Chen et al. also reported that glycyrrhizin exhibited anti-CoV activity against 10 strains of SARS-CoV in fetal rhesus kidney-4 (fRhK-4) and Vero E-6 cell lines by neutralization test (IC_50_ > 400 µM; CC_50_ > 400 µM) [59].

**Figure 1:**
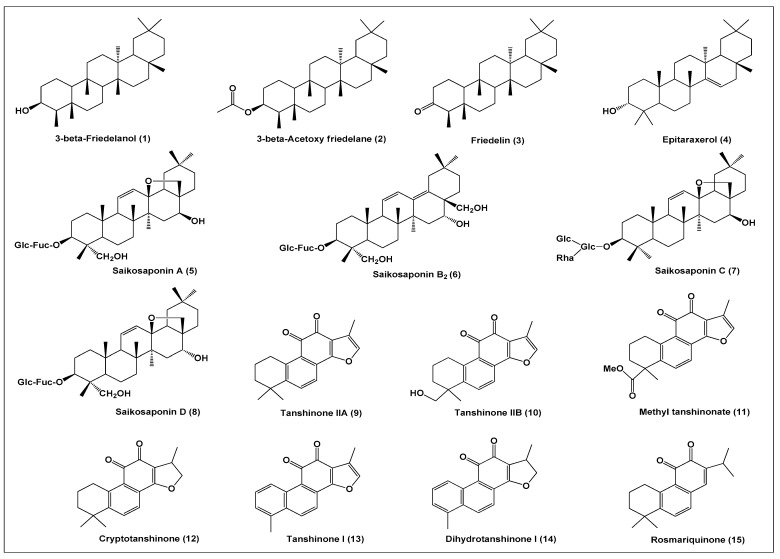

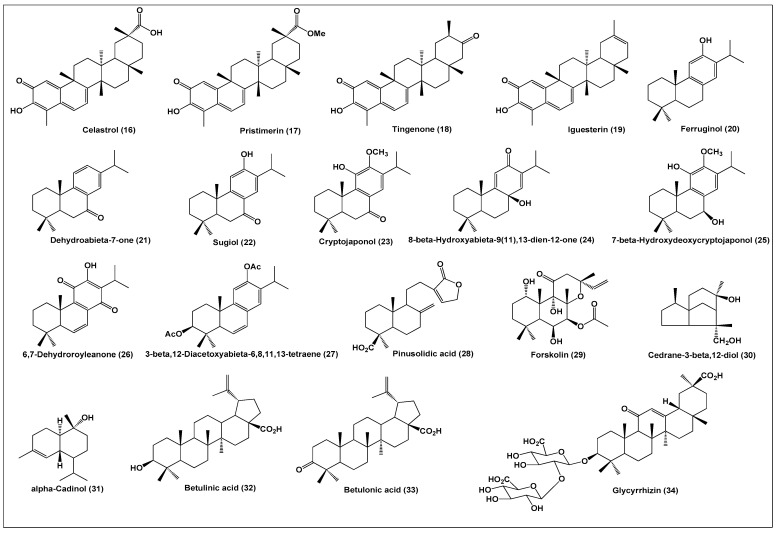
Chemical structure of bioactive terpenes against coronavirus.

**Figure 2:**
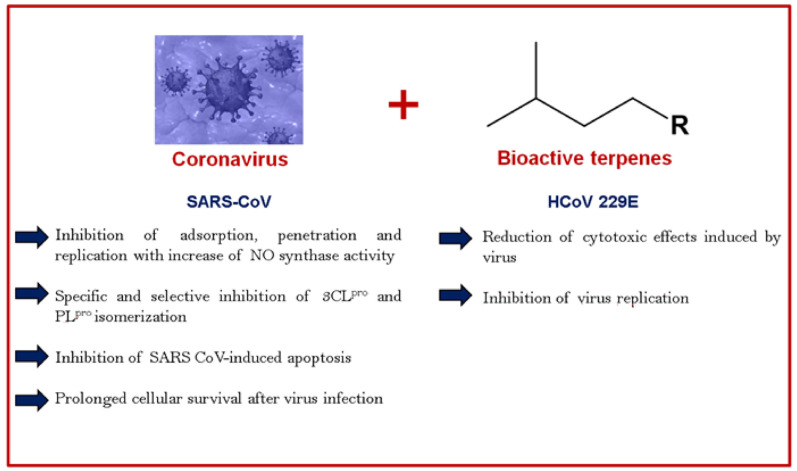
Main mechanisms of action of terpenes against coronaviruses (CoVs).

Moreover, glycyrrhizin was bioactive against the varicella-zoster virus [60], herpex simplex virus [61], hepatitis C virus [62], and dengue virus [63]. The chemical structures of the compounds are shown in Figure 1. Table 1 shows the anti-CoV activity of terpenes, and Table 2 shows the antiviral activity of terpenes against viruses other than CoVs. Figure 2 presents the main mechanisms of action of terpenes against CoVs.

Although the bioactive compounds discussed belong to the same chemical class, there are a diversity of carbonic skeletons and functional groups in chemical structures, such as hydroxyls, carboxylic acids, carbonyls, esters, and ether, including sugar substructures. In addition, antiviral tests were performed under different experimental conditions and/or biological models. Therefore, it is not possible to establish a structure–activity relationship. However, some compounds have high antiviral potency and good SI, such as saikosaponin B2 (1.7 ± 0.1 µM; SI = 221.9), 8β-hydroxyabieta-9 (11), 13-dien-12-one (1.47 µM; SI = > 500), 3β, 12-diacetoxyabieta-6,8,11,13-tetraene (1.57 µM; SI = 193.2), and betulonic acid (0.63 µM; SI = 177.8). Therefore, the compounds presented can be used as prototypes to advance the search for new candidates for anticoronavirus drugs.

### Molecular Modeling

Despite that some of the above-listed terpenes have been assayed against the SARS-CoV virus and its enzymes, many of them lack studies regarding the inhibition of any of the M^pro^ and PL^pro^ enzymes in neither the SARS-CoV nor the SARS-CoV-2 viruses. Regardless of the high similarity of both proteases in the two viruses, it is worth exploring if the already known SARS-CoV protease inhibitors could maintain their inhibition capabilities against the SARS-CoV-2 proteins. The docking results of the 34 terpenes under investigation are presented as Supplementary Materials in Tables S1 (M^pro^) and S2 (PL^pro^). Molecular docking solutions were found for all the 34 compounds in both receptors and filtered following the procedure described in the Methods section (Section 4) to keep those most probable for each compound. This filtering step included the visualization of the predicted ligand poses to discard those falling outside the binding cavity and sets of highly similar conformations of the same compound. This resulted in 65 and 49 potential terpene–M^pro^ and terpene–PL^pro^ complexes, respectively. The larger number of complexes obtained for M^pro^ can be due to its larger binding cavity compared to PL^pro^.

According to the consensus scoring criterion employed for selecting the most probable binding modes of the ligands, compounds **13**, **7**, **14**, **24**, and **9** scored the best in M^pro^. On the other hand, compounds **29**, **28**, **2**, **29**, and **34** are predicted as the less probable M^pro^ inhibitors in docking calculations. The same analysis for PL^pro^ reveals that compounds **19**, **27**, **10**, **13**, and **14** scored the highest, while the worst-scoring PL^pro^ inhibitor candidates are **8**, **17**, **5**, **6**, and **34**. It has been shown that the postprocessing of molecular docking predictions using the estimation of the free energies of binding from molecular dynamics (MD) simulations increases the reliability of structure-based modeling workflows [27,64]. For this reason, MD simulations and MM-PBSA calculations aiming at the more accurate stability evaluation of the docking predicted ligand–receptor complexes were performed as described above.

**Figure 3:**
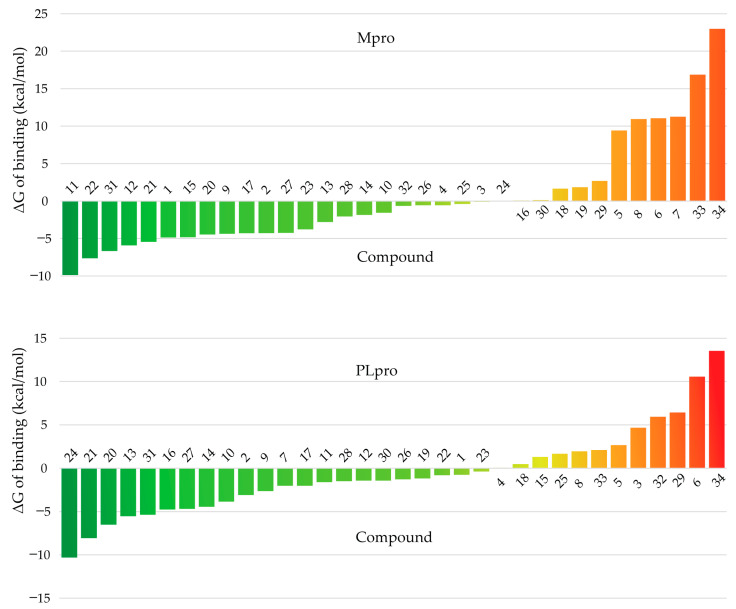
Predicted free energies of binding of the investigated compounds to the M^pro^ (top) and PL^pro^ (bottom) enzymes of SARS-CoV-2. Compounds are ranked, for each target, from lowest (best) to highest (worst) ΔG of binding.

The detailed results of the free energies of binding calculated for the evaluated terpenes against M^pro^ and PL^pro^ are provided in the Supplementary Materials Tables S3 and S4, respectively, and summarized in Figure 3. Only seven compounds (**5**, **6**, **8**, **18**, **29**, **33**, and **34**) out of 34 are predicted with positive free energies of binding to the two targets, which indicates that the predicted complexes with M^pro^ and PL^pro^ are unfeasible. Twenty-three compounds are predicted to possess negative values of ΔG of binding to M^pro^, and 22 fulfill the same criterion for PL^pro^. This suggests that some terpenes may serve as promising compounds for the development of inhibitors of M^pro^ and PL^pro^. Notably, when the lists of the five top-scoring compounds provided by the docking and MM-PBSA calculations are compared, it is seen that only compound **13** appears in both lists for PL^pro^.

**Figure 4:**
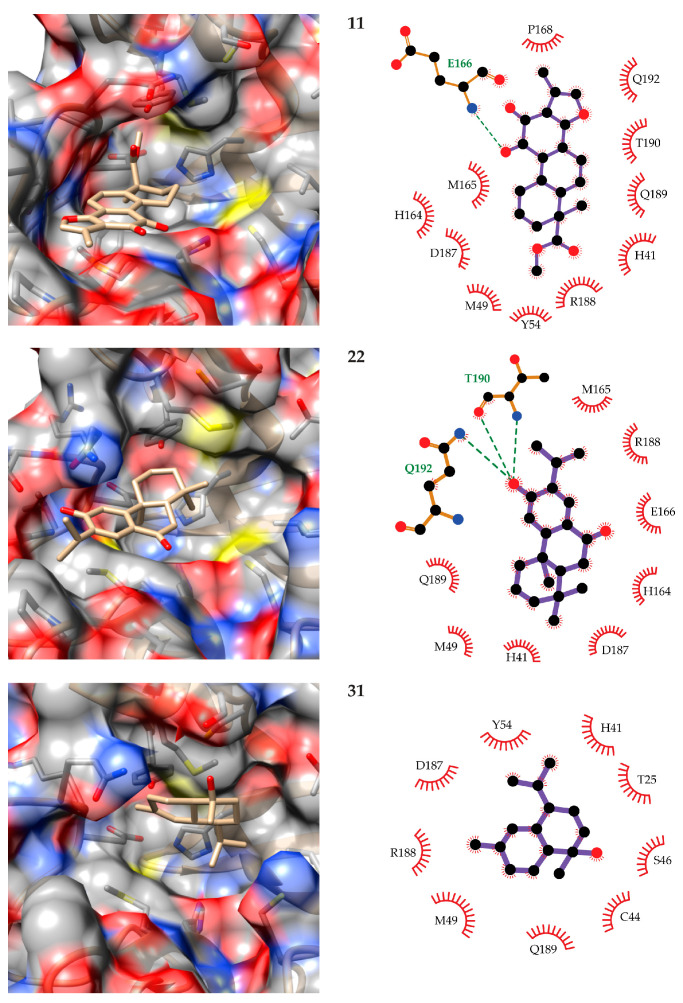
Predicted binding modes of **11** (Methyl tanshinonate, top), **22** (Sugiol, center), and **31** (α-Cadinol, bottom) to the SARS-CoV-2 M^pro^ (left) and diagrams of the observed ligand–receptor interactions (right). The receptor surface is colored by atom type: gray for carbon, red for oxygen, yellow for sulfur, and blue for nitrogen. All atoms are represented only for amino acids forming hydrogen bonds with the ligands in the interaction diagrams. In these diagrams, hydrogen bonds are represented by dashed lines, carbon atoms are represented in black, and the rest of the atoms are colored as in the left images.

Given that the calculation of the free energies of binding from MD simulations provide more accurate estimations of the feasibility of ligand–receptor complexes than molecular docking alone, from here on all discussion will be based on the results presented in Figure 3. According to these, compounds **11** (methyl tanshinonate), **22** (sugiol), and **31** (α-cadinol) are the top three candidates for M^pro^ inhibition among the 34 terpenes evaluated. The predicted orientation of these compounds in the M^pro^ binding cavity as well as the diagrams of their interactions with the receptor are depicted in Figure 4. The structure used for depiction corresponds to the centroid of the largest cluster resulting from the clustering of the ligand conformations along the 100 MD snapshots used for MM-PBSA calculations. The figures were produced with UCSF Chimera [65] and LigPlot+ [66]. Only the interactions observed in at least 50% of the analyzed MD snapshots are represented in the figures, and the interaction frequencies were analyzed with Cytoscape [67].

The predicted conformations of these compounds to M^pro^ show that compounds **11** and **22** bind to the same region of the pocket that comprises its S2, S3, and S4 subcavities [22]. On the other hand, **31** only occupies the S2 subregion. None of the three compounds interact with the catalytic C145 in more than 50% of the analyzed MD snapshots. Compound **31** occasionally interacts (in 26% of the snapshots) with this residue. In contrast, the three ligands directly interact with the catalytic H41 amino acid, thus potentially blocking the access of the substrates to it. Compound **11** is predicted to hydrogen bond the side chain of E166 in most of the studied snapshots, while less frequent interactions of this type are predicted with Q189 and Q192. This ligand forms a network of hydrophobic and van der Waals interactions with M^pro^ that include, besides the aforementioned residues, M49, Y54, H164, M165, P168, D187, R188, and T190.

The second-best candidate for M^pro^ inhibition, compound **22**, is predicted to engage in hydrogen bonding through its hydroxyl substituent to the side chains of T190 and Q192, as well as to the backbone of the former. In addition, one hydrogen bond is observed between the ligand carbonyl group and the backbone of E166 in 47% of the analyzed MD snapshots. The rest of the contacts of this compound with M^pro^ are mainly with H41, M49, H164, M165, D187, R188, and Q189. The overlap of the lists of residues interacting with compounds **11** and **22** highlights their highly similar binding modes to M^pro^. Finally, compound **31** is predicted to make contact with T25, H41, C44, S46, M49, Y54, D187, R188, and Q189. Although no hydrogen bond is predicted between this compound and M^pro^ in most of the analyzed snapshots, this type of interaction is observed in 46% of them with Q189.

**Figure 5:**
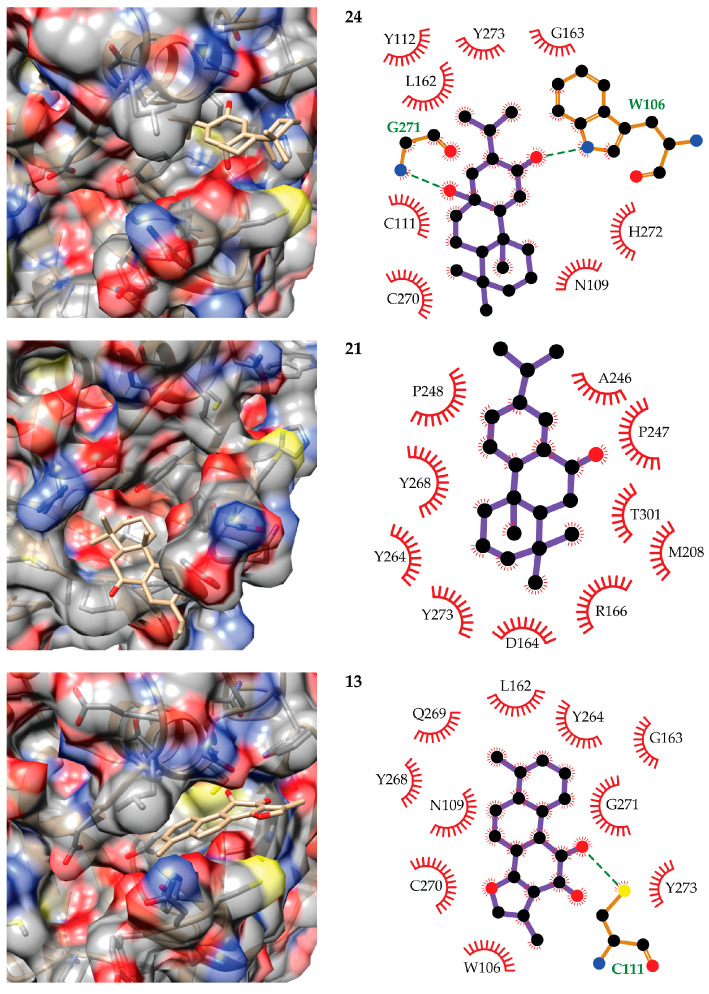
Predicted binding modes of **24** (8-β-hydroxyabieta-9(11),13-dien-12-one)**, 21** (dehydroabieta-7-one), and **13** (tanshinone I, bottom) to the SARS-CoV-2 PL^pro^ (left) and diagrams of the observed ligand–receptor interactions (right). The receptor surface is colored by atom type: gray for carbon, red for oxygen, yellow for sulfur, and blue for nitrogen. All atoms are represented only for amino acids forming hydrogen bonds with the ligands in the interaction diagrams. In these, hydrogen bonds are represented by dashed lines, carbon atoms are represented in black, and the rest of the atoms are colored as in the left images.

Regarding PL^pro^, the best inhibitory terpenes are compounds **24** (8-β-hydroxyabieta-9(11),13-dien-12-one)**, 21** (dehydroabieta-7-one), and **20** (ferruginol). These compounds share high structural similarity, and the predicted binding mode of compound **20** is almost identical to that of **21**. To get additional insights into the possible mechanism of action of terpenes against PL^pro^, we investigated the theoretical complexes of compounds **24, 21**, and **13** (Tanshinone I, ranked in the fourth position) with this SARS-CoV-2 enzyme. The predicted poses of these compounds in the PL^pro^ active site and the diagrams of their interactions with the receptor are depicted in Figure 5.

The active site of PL^pro^ can be subdivided into four regions S1–S4 associated with substrate recognition. Among these, S2 is a narrow channel that connects S1 with S3 and S4 [3]. The terpenes herein evaluated are bulky compounds and no ligand conformation could be obtained that simultaneously bind to the S1 and S3/S4 subsites. Instead, ligand conformations binding to the S1-S2 sites and S2-S3-S4 subpockets were obtained from the modeling workflow. Among the best candidates for PL^pro^ inhibition, compounds **24** and **13** are predicted to bind at the S1-S2 region, whereas compound **21** binds to the S2-S3 subsites. Compound **24** is predicted to hydrogen bond W106 and G271, the former being a critical residue for the stabilization of the negatively charged intermediates at the oxyanion hole during catalysis in SARS-CoV [68]. Less frequent hydrogen bonds are formed (in 24% of the analyzed MD snapshots) with the catalytic C111, although contacts with this residue are predicted in all the snapshots. This compound also directly interacts with H272 that is part of the catalytic triad. The rest of the interactions of compound **24** with the receptor are predicted to occur with N109, Y112, L162, G163, C270, H272, and Y273.

The modeling of compound **21** (and **20**) reveals a binding mode in which it does not interact with any of the PL^pro^ catalytic residues. However, its positioning at the S3-S4 sites would prevent substrate binding and block access to the active site of the enzyme. This ligand is predicted to bind in a region mainly flanked by hydrophobic residues that include A164, R166, M208, A246, P247, P248, Y264, Y268, Y273, and T301. It is interesting to note that compounds **24**, **21**, and **20** are highly similar, but they are predicted to bind to different regions of PL^pro^. This can be explained by their subtle structural differences. While compound **24** is predicted to hydrogen bond the receptor through its carbonyl and hydroxyl substituents, these groups are at different positions in compounds **21** and **20**, which hinders the formation of these hydrogen bonds with the later compounds. The loss of these two hydrogen bonds with the receptor causes a large decrease in the ΔG of the binding of compounds **21** and **20** to PL^pro^.

The last candidate inhibitor of PL^pro^ is compound **13**. Its predicted binding mode overlaps with that of compound **24**, with the possibility of directly hydrogen bonding the side chain of the catalytic C11 amino acid. Furthermore, it forms hydrogen bonds with the side chain of W106 in 44% of the selected MD snapshots. The network of contacts that this compound makes with the receptor is completed by N109, L162, G163, Y264, Y268, Q269, C270, G271, and Y273. Another interesting result derived from our calculations is that some of the evaluated terpenes could have dual M^pro^-PL^pro^ inhibition activity. This is the case of compounds **21** and **31** that are predicted with ΔG < −5 kcal/mol for both receptors. The first of these ranks fifth and second among all molecules according to their predicted free energies of binding to M^pro^ and PL^pro^, respectively. Likewise, **31** ranks third in the M^pro^ and fifth in the PL^pro^ rankings.

The predicted binding modes of the top candidates identified for the inhibition of the SARS-CoV-2 proteases provide useful insights for their future modification with the objective of improving their affinity with the receptors. For instance, none of compounds **11**, **22**, and **31** exploits the S1 subpocket of M^pro^. The introduction of modifications capable of occupying this region and complementary with the receptor could improve the stability of the predicted complexes. Given the number of amino acids’ side chains capable of donating and/or accepting hydrogen bonds in the large binding cavity of M^pro^, it is possible to modify its candidate inhibitors with substituents favorably positioned to hydrogen bond the receptor. Thus, more stable complexes could be obtained. Similar analyses are also possible for the PL^pro^ enzyme.

No experiments have been devoted so far to study the inhibition of the SARS-CoV-2 M^pro^ and PL^pro^ enzymes by terpenes. However, some of the compounds herein studied have been evaluated against the highly similar M^pro^ and PL^pro^ enzymes of the SARS-CoV virus [39,50,69,70,71]. Our results are in line with this previously published evidence. Methyl tanshinonate (**11**), which ranked first as an M^pro^ inhibitor candidate, is a confirmed inhibitor of this enzyme in SARS-CoV (IC_50_ = 21.1 µM) [39]. The second and third in this list are Sugiol (**22**) and α-Cadinol (**31**), which were screened for their SARS-CoV M^pro^ inhibitory activity [50] and were found inactive at concentrations below 100 µM. In the same report, ferruginol (**20**), which is highly similar to Sugiol and ranks eighth in our list of potential M^pro^ inhibitors, was also assayed with the same result as compounds **22** and **31**. However, a posterior re-evaluation of compound **20** concluded that it inhibits the enzymatic activity of the SARS-CoV M^pro^ enzyme with IC_50_ = 49.6 µM [71]. Unfortunately, sugiol (**22**) and α-Cadinol (**31**) were not re-assayed in these more recent experiments. In light of these data, we believe that it is worth re-evaluating these compounds against the SARS-CoV-2 M^pro^ enzyme.

Among our best SARS-CoV-2 PL^pro^ inhibitor candidates, tanshinone I (**13**) is a confirmed inhibitor of this enzyme in SARS-CoV with IC_50_ = 8.8 µM [39]. The inhibition of PL^pro^ by our first two candidates against this enzyme, 8-β-hydroxyabieta-9(11),13-dien-12-one (**24**) and dehydroabieta-7-one (**21**), has been assayed in neither the SARS-CoV nor the SARS-CoV-2 viruses. However, their inhibition of the SARS-CoV virus has been confirmed through some mechanism not involving action on the M^pro^ enzyme [50]. Our results suggest that the inhibition of PL^pro^ might be the antiviral mechanism of action of these compounds against coronaviruses.

## 4. Conclusions

This study provides a detailed compilation and evidences that plant terpenes and their derivatives must be considered as promising sources for the discovery of effective anti-CoV agents, which can be used as treatments or as adjuvants to conventional COVID-19 therapies. The structural diversity of the investigated compounds makes it difficult to establish a structure–antiviral activity relationship. However, the most promising compounds can be used as prototypes for the discovery of effective drugs against CoVs. In addition, further investigations are needed to establish pharmacokinetic and pharmacodynamic parameters for these compounds. Computational models that included a total of 1.14 µs time of molecular dynamics simulations lead to the identification of promising terpenes for the inhibition of the proteases from the SARS-CoV-2 virus. Methyl tanshinonate (**11**), Sugiol (**22**)**,** and α-Cadinol (**31**) are predicted as the best candidates for M^pro^ inhibition, while 8-β-hydroxyabieta-9(11),13-dien-12-one (**24**), Dehydroabieta-7-one (**21**), and Tanshinone I (**13**) are better positioned as candidate inhibitors of PL^pro^ of the SARS-CoV-2 virus. Interestingly, some of the studied chemicals have the potential to inhibit both protease enzymes. Altogether, our results show that terpenes and their derivatives should be considered in the search for therapeutic alternatives against the SARS-CoV-2. Finally, this class of compounds can be promising chemical scaffolds in the discovery of lead compounds to feed the anti-SARS-CoV-2 drug discovery pipelines.

## Figures and Tables

**Table 1:** Terpenes and derivatives tested against in vitro coronavirus infection models, their main results, and mechanism of action.

**Compound**	Experimental Model	Cellular CytotoxiCity Concentration (CC_50_)	Inhibitory Concentration (IC_50_)	Selectivity Index (SI)	Antiviral Effect	Reference
3-β-Friedelanol **(1)** 3-β-Acetoxy friedelane **(2)** Friedelin **(3)** Epitaraxerol **(4)**	HCoV 229E-infected MRC-5 Cells: XTT assay	Data not published	Data not published	Data not published	Data not published	[32]
Saikosaponin A **(5)** Saikosaponin B_2_ **(6)** Saikosaponin C **(7)** Saikosaponin D **(8)**	HCoV 229E-infected Vero E6 cells	228.1 ± 3.8 µM 383.3 ± 0.2 µM 121.5 ± 0.1 µM 176.2 ± 0.2 µM	8.6 ± 0.3 µM 1.7 ± 0.1 µM 19.9 ± 0.1 µM 13.2 ± 0.3 µM	26.6 221.9 19.2 13.3	Inhibition of HCoV-229E activity	[34]
Tanshinone IIA **(9)** Tanshinone IIB **(10)** Methyl tanshinonate **(11)** Cryptotanshinone **(12)** Tanshinone I **(13)** Dihydrotanshinone I **(14)** Rosmaraquinone **(15)** Tanshinone IIA **(9)** Tanshinone IIB **(10)** Methyl tanshinonate **(11)** Cryptotanshinone **(12)** Tanshinone I **(13)** Dihydrotanshinone **(14)** Rosmaraquinone **(15)**	SARS-CoV 3CL^pro^ synthesized and expressed in *Escherichia coli* SARS-CoV PL^pro^ synthesized and expressed in *Escherichia coli*		89.1 ± 5.2 µM 24.8 ± 0.8 µM 21.1 ± 0.8 µM 226.7 ± 6.2 µM 38.7 ± 8.2 µM 14.4 ± 0.7 µM 21.1 ± 0.8 µM 1.6 ± 0.5 µM 10.7 ± 1.7 µM 9.2 ± 2.8 µM 0.8 ± 0.2 µM 8.8 ± 0.4 µM 4.9 ± 1.2 µM 30.0 ± 5.5 µM		Specific and selective inhibition of SARS-CoV replication by noncompetitive SARS-CoV 3CL^pro^ and PL^pro^ protease isomerization	[39]
Celastrol **(16)** Pristimerin **(17)** Tingenone **(18)** Iguesterin **(19)**	Fluorescent enzymatic assay Lineweaver–Burk and Dixon plots		10.3 ± 0.2 µM 5.5 ± 0.7 µM 9.9 ± 0.1 µM 2.6 ± 0.3 µM		Reduction of SARS-CoV replication by competitive inhibition of SARS-CoV 3CL^pro^ protease activity	[43]
Ferruginol **(20)** Dehydroabieta-7-one **(21)** Cryptojaponol **(23)** 8β-Hydroxyabieta-9(11),13-dien-12-one **(24)** 6,7-dehydroroyleanone **(26)** 3β,12-Diacetoxyabieta-6,8,11,13-tetraene **(27)** Pinusolidic acid **(28)** Forskolin **(29)** Cedrane-3-β,12-diol **(30)** α-Cadinol **(31)** Betulinic acid **(32)** Betulonic acid **(33)** 7β-hydroxydeoxy-cryptojaponol **(25)**	SARS-CoV-infected Vero E6 cells	80.4 µM 305.1 µM 78.5 µM >750 µM 89.7 µM 303.3 µM >750 µM 674 µM >750 µM 76.8 µM 150 µM 112 µM 127 µM	1.39 µM 4.00 µM >10 µM 1.47 µM 5.55 µM 1.57 µM 4.71 µM 7.5 µM >10 µM 4.44 µM >10 µM 0.63 µM 1.15 µM	57.8 76.3 <7.9 >500 16.2 193.2 >159 89.8 - 17.3 <15 177.8 110.4	Inhibition of SARS CoV-induced infection/ apoptosis and prolonged cellular survival after virus infection	[50]
Glycyrrhizin **(34)** After virus adsorption During and after virus adsorption During virus adsorption Glycyrrhizin **(34)** 18β-Glycyrrhetinic acid (Glycyrrhizin aglicone) Glycyrrhizin **(34)**	SARS-CoV-infected Vero cell SARS-CoV-Vero E-6 cell line (48 and 72 h) SARS-CoV-(fRhK-4) cell line (48 h) SARS-CoV-infected Vero cell	>20,000 mg/L >20,000 mg/L >20,000 mg/L >400 µg/mL 20 ± 5 µM	600 mg/L 300 mg/L 2400 mg/L >400 µg/mL >20 µM	>33 >67 8.3 - 1	Inhibition of adsorption, penetration, and replication of SARS-CoV with an increase of NO synthase activity Anti-SARS-CoV activity by neutralization test	[58] [59] [56]

**Table 2:** Bioactivity of terpenes against other viruses besides the coronavirus.

Compound	Type of Virus/Cell Lines/Animal Model	Concentration/Dose	Antiviral Effect	Reference
3-β-Friedelanol **(1)**	Hepatitis B Virus/HepG2.2.15 cells	88.5 μM	Inhibits 50% of HBeAg secretion	[33]
Saikosaponin A **(5)**	Influenza A vírus (H1N1 PR8, H9N2, and H5N1)/A549 cells	1.98, 2.21 and 2.07 Μm, respectively	Inhibits 50% of virus replication	[35]
Saikosaponin B_2_ **(6)**	Hepatitis C Virus/Huh-7 cells	50 μM	Inhibits the entry of HCV in primary human hepatocytes, preventing viral attachment and inhibiting viral entry/fusion	[36]
Saikosaponin C **(7)**	Hepatitis B Virus/HepG2.2.15 cells	11 μg/mL 13.4 μg/mL	Inhibits 50% of HBsAg secretion Inhibits 50% of HBV DNA expression	[37]
Saikosaponin D **(8)**	Herpes Simplex Virus and Measles Virus/Vero cells	5 μM	Direct inactivation of virus effects, significantly inhibiting virus replication	[38]
Tanshinone IIA **(9)**	Human immunodeficiency virus-1/TZM-bl cells	10 μM	Reversed Tat-induced reactive oxygen species production through the upregulation of nuclear factor erythroid-derived 2-like2 expression	[40]
	Coxsackievirus B3/BALB/c mice	20 mg/kg (i.p)	Improved hemodynamic parameters, increased levels of IL-4 and IL-10, and decreased IFN-γ and IL-2 levels	[41]
Cryptotanshinone **(12)**	Influenza A virus/293T-IAV-Luc cell	10 μM	Inhibits 97.6% of virus replication	[42]
Pristimerin **(17)**	Human cytomegalovirus/human embryonic lung fibroblast line (MRC-5)	0.53 μg/mL	Inhibits 50% of the synthesis of viral DNA, without affecting cell growth, and reduces immediate early antigen production	[47]
Celastrol **(16)**	Dengue virus-1, -2, -3, and -4/Huh-7 cells	0.19, 0.12, 0.16, and 0.17 μM, respectively	Inhibits 50% of RNA virus replication, induces antiviral IFN-α gene expression	[44]
	Hepatitis C virus/Ava5 cells	0.7 μM	Fully inhibits HCV RNA and protein synthesis via the induction of the c-Jun-N-terminal kinase/nuclear factor erythroid 2-related factor 2 and heme oxygenase 1 axis	[45]
	Human immunodeficiency virus-1/human promonocytic U937 cell line	0.15 μM	Inhibits HIV transcription via a NF-κB-independent mechanism	[46]
Betulinic acid **(32)**	Hepatitis C virus/Ava5 cells Hepatitis B virus/primary hepatocytes from mice Influenza A (PR/8 virus)/A549 cells Herpes Simplex Virus Type-2/Vero cells	40 μM 15 μg/mL 50 μM 1.6 μM	Reduces Hepatitis C virus replication by suppressing the expression of COX-2 COX-2 Inhibited HBV replication by reducing oxidative stress and mitochondrial dysfunction through downregulation of manganese superoxide dismutase expression Attenuated necrosis, numbers of inflammatory cells, and pulmonary edema induced by virus Inhibits 50% of virus replication	[51] [52] [53] [54]
Glycyrrhizin **(34)**	Varicella-zoster virus/human embryonic fibroblast	0.71 mM	Reduced the number of loci by 50%	[60]
	Herpes simplex virus/Wistar rats	1 mg/kg (i.p.)	Attenuate inflammatory responses through inhibition of intercellular adhesion	[61]
	Hepatitis C Virus/Huh-7 cells	14 μg	Inhibits the expression of the HCV 3a core gene at mRNA and protein levels	[62]
	Dengue virus-2/Vero E6 cells	8.1 μM	Inhibited virus cytopathic effect and infectivity by 50%	[63]

## Data Availability

The data presented in this study are available as Supplementary Materials. These include Tables S1 and S2 with the detailed docking results for the 34 compounds against the M^pro^ and PL^pro^ enzymes, respectively. Tables S3 and S4 contain the full results of the MM-PBSA calculations performed for all the studied complexes between the compounds and the M^pro^ and PL^pro^ enzymes, respectively. The predicted complexes of compounds **11**, **22** and **31** with M^pro^ and of **13**, **21** and **24** with PL^pro^ are provided in PDB format within the SM_Structures.zip file.

## Supplementary Materials

The following are available online at https://www.mdpi.com/2218-273X/11/1/74/s1, Table S1: Docking results of the investigated compounds against the SARS-CoV-2 Mpro enzyme, Table S2: Docking results of the investigated compounds against the SARS-CoV-2 PLpro enzyme, Table S3: Estimated free energies of binding of the ligand-Mpro complexes selected from docking calculations, Table S4: Estimated free energies of binding of the ligand-PLpro complexes selected from docking calculations.

## Abbreviations

3CLpro or Mpro	3-Chymotrypsin-like protease or Main protease
ACE2	Angiotensin-converting enzyme 2
CC50	Cytotoxic concentration of the compound that reduced cell viability to 50%
CoVs	Coronaviruses
DPP4	Dipeptidyl-peptidase 4
EC50	Effective concentration of compound needed to inhibit 50% of cytopathic effect
fRhK-4	Fetal rhesus kidney-4
GL	Glycyrrhizin
HBV	Hepatitis B virus
HCoV	Human coronavirus
HCV	Hepatitis C virus
IBV	Infectious bronchitis virus
MD	Molecular dynamics
MERS-CoV	Middle East Respiratory Syndrome-CoV
MHV	Mouse hepatitis virus
ORF1a	Open reading frame 1a
ORF1b	Open reading frame 1b
PLpro	Papain-like protease 2
PP1a	Polyprotein ia
PP1b	Polyprotein 1b
RdRp	RNA-dependent RNA polymerase
SARS-CoV	Severe Acute Respiratory Syndrome-CoV
SARS-CoV-2	Severe Acute Respiratory Syndrome-CoV-2
SI	Selectivity index
TGEV	Transmissible gastroenteritis virus
