# Supplementary material for: Bioactive Terpenes and Their Derivatives as Potential SARS-CoV-2 Proteases Inhibitors from Molecular Modeling Studies
Source: Biomolecules. 2021 Jan 7;11(1):74. doi: 10.3390/biom11010074 (PMC7825698; doi:10.3390/biom11010074)
Supplement: Supplementary file 1 [file biomolecules-11-00074-s001.pdf]

Supplementary Tables for Article:

Bioactive Terpenes and their Derivatives as Potential SARS-CoV-2 Proteases Inhibitors from Molecular Modeling Studies

Table TS1. Docking results of the investigated compounds against the SARS-CoV-2 Mpro enzyme 2

Table TS2. Docking results of the investigated compounds against the SARS-CoV-2 PLpro enzyme 4

Table TS3. Estimated free energies of binding of the ligand-Mpro complexes selected from docking calculations..... 6

Table TS4. Estimated free energies of binding of the ligand-PLpro complexes selected from docking calculations..... 8

Table TS1. Docking results of the investigated compounds against the SARS-CoV-2 Mpro enzyme

Compound	Conformer	CHEMPLP		GoldScore		ChemScore		ASP		Consensus Z-Score
		Score	Z-Score	Score	Z-Score	Score	Z-Score	Score	Z-Score	
1	1	60.53	2.47	-23.37	1.11	29.05	2.16	17.41	1.24	1.75
	2	53.17	0.76	-28.20	0.06	27.90	1.73	18.71	1.75	1.07
2	1	57.84	2.34	-27.32	0.59	25.11	1.26	11.42	-0.89	0.83
3	1	52.38	0.99	-7.78	0.49	28.05	1.68	20.90	2.13	1.33
	2	58.11	2.19	-4.38	1.01	25.06	0.27	16.31	0.67	1.04
4	1	59.08	2.37	15.92	1.49	26.26	1.51	20.33	1.92	1.82
	2	53.98	1.21	6.00	0.28	26.22	1.49	18.30	1.27	1.06
5	1	75.90	2.08	-37.28	0.54	17.23	2.07	28.05	2.06	1.69
6	1	69.02	0.92	-8.45	1.03	20.05	2.40	30.31	2.68	1.76
7	1	89.47	1.80	-7.05	1.68	21.18	2.66	32.20	1.80	1.98
8	1	68.06	1.30	-26.81	0.77	18.63	1.68	30.53	1.51	1.32
	2	66.82	1.17	-24.07	0.85	17.77	1.54	25.26	0.82	1.10
9	1	57.66	1.17	44.70	1.38	26.79	2.37	28.31	1.06	1.50
	2	63.73	2.57	40.99	0.52	24.31	1.24	28.27	1.05	1.34
	3	57.25	1.07	42.95	0.97	23.26	0.76	30.89	1.87	1.17
10	1	58.56	1.01	42.71	1.35	22.19	1.42	33.24	2.13	1.48
	2	61.53	1.66	41.71	1.15	19.38	0.20	29.80	1.25	1.07
	3	58.28	0.95	42.70	1.35	21.72	1.22	27.79	0.75	1.07
11	1	58.79	1.97	36.74	-0.37	21.22	2.63	30.84	1.36	1.40
	2	52.55	0.28	51.40	2.36	18.48	1.25	31.61	1.59	1.37
	3	56.65	1.39	43.37	0.86	18.40	1.21	31.12	1.44	1.23
12	1	63.21	2.46	45.94	1.42	23.69	0.79	26.45	0.86	1.38
13	1	64.98	2.44	49.42	1.81	26.91	2.03	35.00	1.35	1.91
	2	60.66	1.29	45.53	0.99	24.20	0.89	37.02	1.85	1.26
14	1	58.99	1.72	45.17	1.12	24.74	1.60	34.07	2.02	1.62
	2	58.70	1.64	47.44	1.76	22.57	0.36	33.45	1.79	1.39
15	1	53.99	0.37	41.12	1.03	22.37	1.52	26.59	1.68	1.15
16	1	52.07	0.79	-0.15	0.28	22.95	1.62	25.68	2.38	1.27
17	1	58.44	2.67	6.34	0.70	23.33	1.41	22.52	0.99	1.44
18	1	55.82	1.97	13.91	1.28	24.90	2.28	23.10	1.01	1.64
	2	57.07	2.26	17.04	1.77	20.42	0.38	20.06	0.25	1.17
19	1	58.88	1.50	13.19	0.53	27.94	2.13	27.40	2.02	1.55
20	1	60.99	1.83	31.92	1.45	24.20	1.30	21.99	1.44	1.51
	2	56.84	0.94	29.99	0.92	25.18	1.70	23.57	2.02	1.39
	3	57.30	1.04	34.44	2.13	22.09	0.46	22.85	1.76	1.35
21	1	55.19	0.90	34.60	1.65	24.40	2.17	23.08	1.50	1.55
	2	54.82	0.81	29.77	0.42	22.67	1.35	24.84	2.21	1.20

	3	57.36	1.42	32.52	1.12	22.53	1.28	20.82	0.58	1.10
	1	57.79	1.63	34.11	1.85	22.17	1.09	21.90	0.94	1.38
22	2	56.84	1.37	27.36	0.11	24.49	2.03	23.74	1.53	1.26
	3	56.95	1.40	32.45	1.42	21.25	0.72	23.58	1.48	1.26
	1	59.74	1.55	31.24	1.78	20.16	0.83	24.25	1.55	1.43
23	2	56.25	0.83	32.16	2.01	20.83	1.10	24.05	1.48	1.35
	3	59.88	1.58	31.23	1.78	19.61	0.61	22.45	0.94	1.23
24	1	66.99	3.38	36.31	1.84	24.53	1.93	27.83	2.78	2.48
25	1	63.11	2.19	33.72	1.44	21.44	0.56	23.79	1.54	1.43
	2	60.09	1.50	26.93	0.79	22.85	1.10	25.95	2.19	1.40
26	1	53.69	1.11	30.59	1.44	22.48	1.69	23.53	1.70	1.48
	2	54.79	1.40	26.85	0.68	21.86	1.44	20.94	0.50	1.01
27	1	63.13	2.21	36.16	1.02	16.11	0.31	25.89	1.84	1.34
	2	59.61	1.51	37.33	1.14	15.91	0.24	24.22	1.37	1.06
28	1	55.42	1.09	13.32	0.14	15.60	1.71	21.79	1.55	1.12
	1	50.65	0.91	10.06	1.33	16.35	1.42	24.45	1.97	1.41
29	2	50.51	0.88	1.01	0.74	14.74	0.82	24.21	1.88	1.08
	3	50.99	0.99	9.70	1.31	13.94	0.52	22.42	1.22	1.01
30	1	50.60	1.86	23.89	1.26	23.60	2.44	20.21	2.51	2.02
	2	50.24	1.76	23.44	1.16	19.19	0.39	16.59	0.81	1.03
31	1	52.98	1.60	21.74	1.27	23.55	2.41	19.80	1.51	1.70
	2	50.80	1.04	22.57	1.42	20.71	1.00	18.90	1.10	1.14
32	1	62.23	2.08	-16.07	0.51	23.34	1.57	19.60	1.69	1.46
	2	55.25	0.86	-8.09	1.22	21.90	1.18	16.44	0.96	1.06
	1	59.82	1.82	-5.31	1.54	24.28	2.08	20.91	1.83	1.82
33	2	59.01	1.68	-4.69	1.63	22.08	1.51	19.72	1.54	1.59
	3	58.94	1.66	-7.05	1.27	18.22	0.50	19.14	1.41	1.21
34	1	60.69	1.70	-25.33	0.88	3.57	0.96	20.32	0.63	1.04

Table TS2. Docking results of the investigated compounds against the SARS-CoV-2 PLpro enzyme

Compound	Conformer	CHEMPLP		GoldScore		ChemScore		ASP		Consensus
		Score	Z-Score	Score	Z-Score	Score	Z-Score	Score	Z-Score	Z-Score
1	1	48.99	1.15	-32.81	1.80	23.39	0.58	17.02	0.58	1.03
	2	54.82	1.74	-39.21	0.09	29.59	1.26	20.85	0.97	1.01
2	1	46.93	1.08	-38.18	0.35	24.04	0.76	18.50	0.62	0.70
3	1	56.03	1.75	-11.80	1.12	29.03	0.94	20.35	0.88	1.17
4	1	45.26	0.83	7.97	1.73	24.58	0.79	17.88	0.60	0.99
5	1	52.46	1.41	-50.84	0.34	6.55	0.83	14.83	0.74	0.83
6	1	43.47	1.09	-55.07	0.38	7.53	0.24	24.49	0.71	0.61
7	1	46.56	1.18	-67.84	0.34	5.85	1.35	37.01	1.86	1.18
8	1	47.47	1.04	-60.31	0.39	6.00	0.93	31.71	0.97	0.83
9	1	52.49	2.27	41.27	2.18	18.40	-0.79	26.55	2.00	1.41
	2	48.26	1.41	41.25	2.17	23.11	1.19	20.51	0.37	1.28
	3	48.32	1.42	42.35	2.34	21.49	0.51	21.77	0.71	1.24
	4	48.71	1.50	26.20	-0.06	24.12	1.61	26.22	1.91	1.24
10	1	55.75	2.34	42.31	0.26	17.26	0.25	31.80	1.66	1.13
	2	51.70	1.58	26.27	0.25	22.53	1.27	27.32	0.99	1.02
11	1	51.09	1.93	30.19	1.07	17.41	0.70	24.13	0.66	1.09
12	1	48.62	1.61	25.09	0.18	24.36	1.59	22.21	0.93	1.08
13	1	54.48	2.72	38.77	0.20	20.29	0.23	31.07	1.93	1.27
	2	49.60	1.52	37.03	0.20	19.82	-0.03	32.81	2.35	1.01
14	1	50.77	1.75	46.84	2.95	17.48	-0.98	34.81	2.49	1.55
	2	50.47	1.69	38.02	1.49	21.67	0.47	33.69	2.28	1.48
	3	52.25	2.07	32.08	0.50	25.15	1.68	23.09	0.23	1.12
	4	48.97	1.36	39.78	1.78	20.60	0.10	26.73	0.94	1.04
15	1	49.59	1.50	27.21	0.18	23.03	2.08	20.63	0.68	1.11
16	1	48.50	1.55	12.81	2.24	14.00	0.59	9.96	0.39	1.19
	2	46.51	1.40	1.23	1.01	18.25	0.95	17.71	0.91	1.07
17	1	39.22	1.46	-1.27	0.26	19.38	1.36	18.56	1.23	1.08
18	1	48.05	1.32	0.52	0.81	23.37	1.01	25.44	1.19	1.08
	2	47.62	1.28	2.81	1.21	25.70	1.23	17.74	0.54	1.06
19	1	62.66	2.42	19.60	2.31	29.07	1.28	19.27	0.69	1.68
20	1	53.53	1.49	21.31	0.19	25.23	1.49	24.01	1.50	1.17
21	1	55.81	1.83	18.71	0.18	26.15	1.69	22.73	1.30	1.25
22	1	52.23	1.85	26.10	0.19	25.10	1.92	26.13	2.19	1.54
23	1	53.60	1.76	17.83	0.19	22.90	1.24	23.81	1.07	1.06
24	1	52.65	1.44	25.98	1.90	21.52	0.86	25.22	1.62	1.45
	2	52.55	1.42	21.19	1.11	22.75	1.14	22.96	1.22	1.22
	3	53.39	1.54	23.43	1.48	20.70	0.67	21.97	1.04	1.18

25	1	50.51	1.44	15.27	0.19	21.46	1.37	20.91	1.16	1.04
26	1	54.29	2.44	20.99	0.19	21.86	1.33	26.33	1.72	1.42
27	1	57.39	2.14	26.84	1.59	23.68	1.33	28.52	1.44	1.63
	2	52.10	1.43	25.64	1.39	18.53	0.72	25.45	1.09	1.16
28	1	55.79	1.27	20.60	1.19	15.71	0.92	20.02	0.90	1.07
29	1	49.09	1.83	-33.31	0.16	19.43	1.62	21.72	0.90	1.13
30	1	57.34	2.06	24.72	1.47	23.82	1.33	21.55	1.12	1.50
31	1	51.90	2.09	15.15	0.85	19.24	0.90	14.32	0.45	1.07
	2	41.49	0.56	19.16	1.52	21.33	1.27	17.41	0.88	1.06
32	1	49.76	1.92	-29.35	0.56	19.52	1.23	15.81	0.64	1.09
33	1	49.39	1.51	-12.72	1.66	18.86	0.94	16.89	1.08	1.30
34	1	48.76	1.13	-60.91	0.41	5.73	1.20	17.01	0.82	0.89

Table TS3. Estimated free energies of binding of the ligand-Mpro complexes selected from docking calculations

Compound	Conformer	MM-PBSA Component							ΔG
		VDWAALS	EEL	EPB	ENPOLAR	EDISPER	ΔG gas	ΔG solv	
1	1	-46.49	-1.01	15.85	-30.29	57.09	-47.50	42.65	-4.85
	2	-50.67	-4.96	23.33	-32.77	60.82	-55.63	51.38	-4.25
2	1	-49.39	-1.39	17.93	-33.24	61.81	-50.77	46.50	-4.27
3	1	-43.19	0.16	16.17	-29.97	56.79	-43.03	43.00	-0.04
	2	-43.19	-3.08	20.33	-27.64	53.49	-46.28	46.18	-0.10
4	1	-47.10	-3.54	22.59	-30.90	58.40	-50.64	50.09	-0.55
	2	-33.02	-4.04	17.18	-21.95	44.09	-37.06	39.32	2.26
5	1	-65.43	-28.09	68.46	-43.73	82.60	-93.52	107.32	13.80
6	1	-63.62	-23.35	57.97	-41.53	79.95	-86.97	96.39	9.42
7	1	-69.41	-38.09	74.86	-45.70	89.38	-107.50	118.54	11.04
8	1	-57.47	-29.15	61.89	-38.15	74.14	-86.62	97.88	11.26
	2	-60.14	-36.76	69.53	-41.56	79.87	-96.90	107.85	10.95
9	1	-32.91	-4.43	16.52	-22.51	41.53	-37.34	35.54	-1.80
	2	-40.09	-4.31	19.31	-25.44	46.17	-44.41	40.04	-4.36
	3	-33.01	-3.70	16.82	-21.94	39.66	-36.71	34.54	-2.17
10	1	-33.41	-5.70	20.49	-21.98	40.14	-39.11	38.65	-0.46
	2	-36.47	-8.20	29.98	-22.93	42.51	-44.67	49.55	4.88
	3	-36.83	-4.75	19.62	-23.44	43.84	-41.58	40.02	-1.56
11	1	-45.45	-8.23	23.76	-27.67	47.75	-53.69	43.84	-9.85
	2	-33.02	-6.55	25.27	-21.67	40.68	-39.57	44.29	4.71
	3	-32.49	-8.47	23.75	-22.58	40.94	-40.96	42.12	1.16
12	1	-40.40	-4.80	18.97	-25.61	45.93	-45.20	39.29	-5.91
13	1	-32.25	-6.78	19.28	-21.50	38.47	-39.03	36.25	-2.78
	2	-32.87	-2.68	17.40	-21.07	38.68	-35.55	35.02	-0.54
14	1	-38.25	-12.15	29.26	-25.48	44.77	-50.40	48.56	-1.85
	2	-32.81	-6.23	20.80	-21.70	39.58	-39.04	38.68	-0.36
15	1	-39.27	-11.96	26.80	-27.34	46.95	-51.23	46.40	-4.83
16	1	-45.13	-9.73	28.47	-29.08	55.56	-54.86	54.95	0.09
17	1	-51.55	-5.83	25.13	-32.84	60.78	-57.38	53.07	-4.30
18	1	-43.58	-5.46	25.89	-28.22	53.89	-49.04	51.57	2.52
	2	-38.04	-6.03	22.02	-24.20	47.92	-44.07	45.74	1.66
19	1	-42.95	-19.73	38.60	-29.57	55.50	-62.68	64.53	1.85
20	1	-37.44	-10.69	23.67	-25.89	45.89	-48.13	43.67	-4.46
	2	-36.96	-2.93	16.28	-25.35	45.56	-39.89	36.50	-3.39
	3	-33.74	-3.21	19.61	-23.20	41.21	-36.95	37.62	0.67
21	1	-37.31	-0.43	16.86	-26.43	45.37	-37.74	35.80	-1.94

	2	-35.50	-4.43	19.22	-24.92	43.41	-39.93	37.72	-2.21
	3	-38.07	-1.70	14.87	-26.66	46.12	-39.78	34.34	-5.44
22	1	-36.46	-3.55	20.42	-25.57	45.24	-40.02	40.09	0.08
	2	-33.37	-4.85	19.81	-24.98	43.49	-38.22	38.32	0.10
	3	-39.99	-10.16	22.65	-26.61	46.49	-50.15	42.52	-7.63
23	1	-43.10	-3.08	20.63	-29.36	51.15	-46.18	42.42	-3.76
	2	-41.22	-2.40	19.42	-27.51	49.57	-43.62	41.48	-2.14
	3	-37.83	-2.48	19.89	-24.93	45.98	-40.31	40.93	0.62
24	1	-38.07	-4.61	22.68	-26.27	46.24	-42.68	42.65	-0.03
25	1	-41.12	-2.18	22.16	-26.91	49.23	-43.30	44.49	1.19
	2	-39.53	-4.55	21.78	-27.38	49.30	-44.08	43.70	-0.38
26	1	-30.99	-4.65	17.67	-22.58	40.39	-35.64	35.48	-0.16
	2	-38.15	-5.48	22.33	-25.06	45.80	-43.63	43.07	-0.56
27	1	-47.05	-8.08	27.78	-32.03	55.16	-55.13	50.90	-4.23
	2	-46.39	-17.40	36.43	-31.13	54.70	-63.79	60.01	-3.79
28	1	-37.09	51.78	-37.43	-25.69	46.39	14.69	-16.73	-2.05
29	1	-37.29	-4.74	22.64	-24.51	46.59	-42.04	44.73	2.69
	2	-39.26	-5.88	27.80	-26.21	51.79	-45.14	53.38	8.24
	3	-43.31	-16.98	40.87	-29.34	53.55	-60.29	65.08	4.79
30	1	-28.57	-12.56	24.97	-20.49	37.71	-41.13	42.19	1.06
	2	-28.50	-5.25	16.23	-18.25	35.90	-33.76	33.88	0.13
31	1	-29.72	-3.42	11.92	-21.01	35.55	-33.14	26.47	-6.67
	2	-31.97	-5.44	16.72	-23.01	39.78	-37.42	33.48	-3.93
32	1	-43.52	76.04	-56.01	-30.72	57.77	32.51	-28.95	3.56
	2	-49.00	77.49	-54.39	-31.66	56.93	28.49	-29.13	-0.63
33	1	-41.93	101.37	-79.64	-28.58	54.42	59.45	-53.79	5.65
	2	-45.52	78.40	-56.65	-30.56	56.54	32.88	-30.67	2.21
	3	-34.37	87.53	-60.28	-23.31	47.29	53.16	-36.30	16.85
34	1	-51.31	226.79	-188.61	-36.62	72.74	175.48	-152.50	22.98

Table TS4. Estimated free energies of binding of the ligand-PLpro complexes selected from docking calculations

Compound	Conformer	MM-PBSA Component							ΔG
		VDWAALS	EEL	EPB	ENPOLAR	VDWAALS	EEL	DELTA G solv	
1	1	-36.64	-0.10	17.81	-23.80	46.15	-36.74	40.16	3.43
	2	-32.74	-9.50	23.79	-23.34	41.04	-42.24	41.49	-0.75
2	1	-30.29	-1.47	11.27	-21.80	39.22	-31.76	28.68	-3.08
3	1	-27.21	-0.47	16.06	-18.44	34.73	-27.69	32.35	4.66
4	1	-41.70	-17.60	36.62	-28.91	51.65	-59.30	59.37	0.06
5	1	-35.42	-13.16	30.82	-24.42	44.84	-48.58	51.24	2.66
6	1	-43.42	-18.70	45.49	-28.56	55.74	-62.11	72.67	10.56
7	1	-49.48	-89.79	107.06	-38.24	68.44	-139.28	137.26	-2.02
8	1	-33.47	-61.00	75.97	-25.31	45.76	-94.47	96.42	1.95
9	1	-35.72	-4.29	23.64	-23.88	40.73	-40.01	40.49	0.49
	2	-35.91	-7.05	23.80	-24.05	40.59	-42.96	40.34	-2.62
	3	-29.34	-4.39	20.48	-20.10	34.17	-33.72	34.56	0.84
	4	-29.53	-5.07	18.86	-20.13	34.62	-34.60	33.35	-1.25
10	1	-34.61	-7.42	25.35	-22.32	38.76	-42.04	41.79	-0.25
	2	-34.04	-18.27	32.74	-23.24	38.95	-52.31	48.45	-3.86
11	1	-30.67	-7.80	20.71	-20.25	36.41	-38.47	36.87	-1.60
12	1	-30.20	-8.64	22.95	-20.78	35.26	-38.84	37.43	-1.42
13	1	-31.57	-4.43	20.53	-21.40	35.77	-36.00	34.90	-1.11
	2	-32.16	-15.85	28.02	-22.22	36.68	-48.02	42.49	-5.53
14	1	-36.06	-12.41	28.31	-23.16	38.87	-48.47	44.02	-4.45
	2	-33.52	-15.80	31.35	-23.04	37.94	-49.32	46.25	-3.07
	3	-28.76	-13.10	27.36	-18.49	31.24	-41.85	40.11	-1.74
	4	-30.21	-8.70	22.94	-20.84	35.22	-38.91	37.32	-1.60
15	1	-23.95	-5.84	17.82	-17.52	30.82	-29.79	31.12	1.33
16	1	-28.15	-35.87	58.71	-21.98	36.44	-64.02	73.16	9.14
	2	-19.10	-63.21	63.41	-16.67	30.81	-82.30	77.54	-4.76
17	1	-30.65	-16.06	27.19	-21.76	39.27	-46.71	44.70	-2.01
18	1	-27.78	0.90	10.68	-19.20	35.89	-26.88	27.37	0.49
	2	-34.33	-6.53	24.59	-23.60	42.33	-40.86	43.32	2.46
19	1	-37.47	-6.79	23.38	-25.82	45.52	-44.25	43.08	-1.17
20	1	-33.34	-3.07	14.57	-23.13	38.45	-36.40	29.89	-6.52
21	1	-33.42	-6.31	16.49	-23.02	38.20	-39.73	31.67	-8.06
22	1	-32.58	-7.05	23.34	-22.42	37.92	-39.63	38.84	-0.79
23	1	-34.49	-7.80	25.46	-23.64	40.11	-42.29	41.93	-0.36
24	1	-30.55	-22.39	29.49	-23.30	36.44	-52.94	42.64	-10.31
	2	-29.71	-4.32	18.72	-21.41	36.00	-34.03	33.31	-0.72

	3	-39.09	-1.27	20.09	-26.58	44.64	-40.36	38.15	-2.22
25	1	-33.89	-3.33	21.95	-23.26	40.17	-37.22	38.86	1.64
26	1	-31.71	-6.01	20.36	-21.40	37.48	-37.72	36.44	-1.28
27	1	-24.20	-5.50	19.45	-17.96	31.78	-29.70	33.27	3.57
	2	-39.52	-5.65	21.34	-28.62	47.76	-45.17	40.49	-4.69
28	1	-26.14	-37.94	47.62	-19.78	34.74	-64.08	62.59	-1.49
29	1	-33.44	-10.19	31.31	-22.52	41.26	-43.64	50.05	6.41
30	1	-27.19	-0.73	12.42	-18.91	33.00	-27.92	26.51	-1.41
31	1	-30.74	-10.28	23.00	-23.74	36.41	-41.02	35.66	-5.36
	2	-25.35	-15.80	25.31	-20.18	32.33	-41.15	37.46	-3.69
32	1	-25.13	-10.58	26.07	-17.99	33.55	-35.70	41.63	5.93
33	1	-25.59	-6.07	18.26	-18.22	33.71	-31.66	33.75	2.08
34	1	-35.07	-68.52	90.95	-24.87	51.05	-103.59	117.13	13.54
